# Changes in the Abundance and Community Complexity of Soil Nematodes in Two Rice Cultivars Under Elevated Ozone

**DOI:** 10.3389/fmicb.2022.916875

**Published:** 2022-06-09

**Authors:** Jianqing Wang, Yunyan Tan, Yajun Shao, Xiuzhen Shi, Guoyou Zhang

**Affiliations:** ^1^Key Laboratory for Humid Subtropical Eco-geographical Processes of the Ministry of Education, Fujian Normal University, Fuzhou, China; ^2^School of Geographical Sciences, Fujian Normal University, Fuzhou, China; ^3^Key Laboratory of Agrometeorology of Jiangsu Province, School of Applied Meteorology, Nanjing University of Information Science & Technology, Nanjing, China; ^4^Jiangsu Key Laboratory of Crop Genetics and Physiology, Agricultural College of Yangzhou University, Yangzhou, China

**Keywords:** climate change, elevated ozone, soil health, soil food web, paddy soil, soil fauna, rice varieties

## Abstract

The atmospheric ozone concentrations have substantially increased in the surface layer over the past decades, and consequently exhibited a strong influence on soil microbial communities and functions. However, the effect of elevated ozone (eO_3_) on the abundance, diversity, and structural complexity of soil nematode communities are elusive under different rice (*Oryza sativa L*.) cultivars. Here, the soil nematode community was investigated in two rice cultivars (Hybrid, Shanyou 63 vs. Japonica, Wuyujing 3) under open-top chambers (OTC) with control and eO_3_ conditions. The results showed that the abundance of soil nematode community was altered by eO_3_, but the responses were dependent on crop cultivars. The eO_3_ decreased the total abundance and simplified the network complexity of the soil nematode community for both cultivars. However, eO_3_ increased the abundance of c-p 4 in Shanyou 63, rather than Wuyujing 3, indicating that the hybrid rice cultivar could tradeoff the adverse impacts of eO_3_ on the functional group of soil nematodes. Similarly, bacterivores belonging to *K*-strategy (c-p 4) increased under eO_3_ in Shanyou 63, suggesting that the soil food web formed a bacteria-dominated channel under eO_3_ for the hybrid rice cultivar. This study shed new light on the critical importance of rice cultivars in shaping the impacts of eO_3_ on the soil micro-food web. Therefore, breeding and biotechnological approaches may become valuable pathways to improve soil health by shaping the community structures of the soil micro-food web in response to climate change in the future.

## Introduction

Ozone is one of the most important atmospheric pollutants in the surface layer, seriously threatening the health of agricultural ecosystems (Tang et al., 2013). Since the industrial revolution, the concentration of ozone in the surface layer has been increasing and attracting extensive interest (Phillips et al., 2009). Previous studies have revealed that elevated ozone (eO_3_) can damage plant leaf tissue and show detrimental effects on food production (Ashmore, 2005). For example, Zhu et al. (2011) suggested that a 25% increase in the ozone concentration caused a significant decrease of 20% in the grain yield of winter wheat in a fully open-air field condition. A meta-analysis aiming at investigating the impacts of eO_3_ on crop yields showed that higher ozone concentrations (*ca* 31–50 ppb) resulted in the 8.9, 9.7, and 17.5% of declines in the yield production of barley, wheat, and rice, respectively (Feng and Kobayashi, 2009). Furthermore, eO_3_ has been demonstrated to inhibit plant photosynthesis and negatively influence carbon allocation from plant to soil (Andersen, 2003; Ainsworth, 2017). Therefore, eO_3_ can not only affect plant growth and crop production but also threaten the belowground microbial community and biodiversity (Agathokleous et al., 2020).

The soil micro-food web is indispensable for providing soil functions and services, such as food security, nutrient dynamic, biodiversity, and soil health (De Vries et al., 2013; Zhu et al., 2020). The soil nematode community serving as an essential component of the soil micro-food web is one of the most widely distributed invertebrates in global terrestrial ecosystems (Bongers and Ferris, 1999; Van Den Hoogen et al., 2019). Soil nematodes can simultaneously occupy different trophic levels and act as critical bio-indicators for predicting climate change in agroecosystems (Wang et al., 2021). However, large uncertainty exists regarding the influence of eO_3_ on the soil nematode community. Because of the toxic effect of ozone, eO_3_ shows an adverse impact on the abundance and diversity of soil nematodes in cropland fields (Bao et al., 2014; Zhang et al., 2021). A previous study found that the functional diversity of the soil nematode community was sensitive to eO_3_, and the diversity index decreased whereas the dominance index increased under eO_3_ in the open-top chambers (OTCs) for soybean plants (Bao et al., 2014). Li et al. (2015) showed that the legacy effect of eO_3_ led to a significant decrease in the number of bacterivores and fungivores, while the total abundance of soil nematodes was increased by eO_3_ in winter wheat fields (Li et al., 2015).

Plant cultivars can greatly contribute to the variable effects of eO_3_ on soil nematode community, mainly due to the differences in the quantity and quality of plant resources (Li et al., 2012; Zhang et al., 2021). Several studies have verified that crop cultivars exhibited different sensitivity to eO_3_ stress (Shi et al., 2009; Ainsworth, 2017). In a rice-wheat rotation system, wheat cultivars alleviated the residual effects of eO_3_ on soil fungivores (Li et al., 2016). Li et al. (2012) found that the impact of eO_3_ on soil nematode community was more sensitive in ozone-tolerant cultivars than in ozone-sensitive cultivars in winter wheat fields. Rice has been considered vulnerable to eO_3_ compared with other major crop species (Feng and Kobayashi, 2009). The hybrid Indica cultivar, which can express the *Bt* on gene to resist the pests and disease invasion, has been widely cultivated to increase the yield (Tu et al., 2000). However, greater reductions in the leaf photosynthesis and grain yield through oxidative damage to cells have been found in the hybrid Indica cultivar exposed to eO_3_ when compared with conventional cultivars (Xu et al., 2007; Pang et al., 2009). Moreover, the tropospheric ozone concentration could rise 40–60% by the end of the 21st century, resulting from a dramatic increase in the emissions of O_3_ precursors (e.g., VOCs and NOx) (Meehl et al., 2007). To date, the response and underlying mechanism of soil nematode community to eO_3_ remain unclear under different rice cultivars.

The objective of this study is therefore to identify the effects of eO_3_ on the abundance, diversity, and structural complexity of soil nematode community under different rice cultivars through an OTCs experiment. The changes in the ecological environment and agricultural production in response to eO_3_ were explored simultaneously. We hypothesized that (1) eO_3_ would reduce the abundance and diversity of soil nematodes and simplify the structural complexity of the soil micro-food web, due to decreased plant biomass and soil food resources; and (2) soil nematode community would be more susceptible to eO_3_ under the conventional cultivar compared with hybrid Indica cultivar, owing to the relatively low crop biomass or plant C resources input to the soil associated with the conventional cultivar.

## Materials and Methods

### Site Description

The experimental site is located at Jiangdu, in Jiangsu Province (119°43′E, 32°25′N), China. The site belongs to a subtropical monsoon anmate, with the average precipitation and temperature of 1131.3 mm and 16.2°C, respectively, from 2009 to 2018, respectively. The soil is classified as Gleyic Stagnic Anthrosol. The physicochemical properties of topsoil (0–15 cm) were as follows: soil pH 7.05, total carbon (TC) 6.94 g kg^−1^, and total nitrogen (TN) 1.05 g kg^−1^.

### Experimental Design

This experiment was performed in six open-top chambers (OTCs) (i.e., three control conditions, CK vs. three elevated ozone, eO_3_). The octagonal OTCs of 2.3 m in height and 4.8 m in diameter had aluminum alloy frames covered with walls of transparent tempered glass. In this study, an ozone generator (HY003, Chuangcheng Co., Jinan, China) was used to produce ozone, and then the produced gas was mixed and exchanged by a high-power fan (2200 w, CX125, Quanfeng Co., Shanghai, China). An ozone analyzer (Model 49i, Thermo Scientific, USA) was used to monitor and record the ozone concentrations at the plant canopy in the chambers. The flow of compressed oxygen and ozone concentrations was controlled by a mass flow meter according to actual and target ozone concentrations in the OTCs (Shang et al., 2021). The ozone fumigation began on 25 July 2020, with a daily duration between 8:00 a.m. and 6:00 p.m. The average ozone concentrations were 39.6 ± 1.9 ppb and 87.3 ± 0.9 ppb for CK and eO_3_ chambers, respectively.

Rice cultivars of Shanyou 63 (hybrid Indica cultivar) and Wuyujing 3 (conventional japonica cultivar) have commonly been planted in this area. In this study, rice seeds were sown on 20th May 2020. Rice seedlings were transplanted into round pots (31 cm in height 22 cm in diameter, *ca* 15 kg soil) on 2nd July 2020, each pot had 3 hills, and then all pots were transferred to the chambers. In total, each treatment had three OTCs replicates, and each OTCs had 10 round pots, i.e., 5 round pots per cultivar (Shang et al., 2021).

### Sample Collection and Analysis

After rice harvest, five-round pot soils were collected and then mixed as a sample. A total of 12 soil samples (2 ozone levels × 2 cultivars × 3 replicates) were collected. After the removal of all roots and plant residues, all samples were stored at 4°C until further analysis.

Soil microbial biomass carbon (MBC) and nitrogen (MBN) were extracted with 0.5 mol L^−1^ K_2_SO_4_ after fumigation with ethanol-free chloroform and determined by a multi N/C Analyzer (TOC Analyzer, Germany), the differences in fumigated samples were used to calculate soil microbial biomass with coefficients of 0.38 (MBC) and 0.45 and (MBN), respectively. Soil dissolved organic carbon (DOC) was analyzed by a multi-N/C Analyzer (Jena TOC Analyzer, Germany) (Wu et al., 1990). Soil dissolved organic nitrogen (DON) was calculated based on soil contents of NH${}_{4}^{+}$-N, NO${}_{3}^{-}$-N, and TN. Soil NH${}_{4}^{+}$-N and NO${}_{3}^{-}$-N concentrations were extracted with 2 mol L^−1^ KCL, and then were analyzed through a flow injection auto-analyzer (Skalar, The Netherlands). Soil pH was determined by 1/2.5 (soil/water ratio) with Mettler-Toledo pH (Lu, 2000).

### Soil Nematode Analysis

Soil nematodes were extracted using a modified Baermann funnel as previously described (Wang et al., 2019). The abundances of soil nematodes were quantified based on a Motic microscope (40 × and 400 ×). The genera of soil nematodes were identified using 150 randomly selected individuals per soil sample by a Motic microscope. The trophic groups of the nematode community were assigned to bacterivores (BF), fungivores (FF), herbivores (PF), and omnivores-predators (OP), and values of 1–5 *c-p* referred to http://Nemaplex.ucdavis.edu. The abundance of soil nematode was converted to the individuals per 100 g of dry soil. The functional groups of soil nematodes were assigned to colonizers (c) and persisters (p) based on the c-p scale ranging from 1 to 5 (Bongers, 1990). The nematodes of c-p 1 resemble the *r*-strategy and occur in highly disturbed conditions. On the other hand, the nematodes of c-p 5 resemble the *K*-strategy, which is featured by high sensitivity and low fecundity (Neher and Darby, 2009). Soil nematodes were assigned to different functional groups combining the c-p class and the trophic groups of the soil nematode genus (Ferris et al., 2001). The Shannon Diversity Index (H') was used to calculate the diversity of soil nematode communities.

### Statistical Analysis

All data were collated in Microsoft Excel 2016 and expressed as the mean of the three replicates plus/minus the standard deviation. All statistics were performed by R and IBM SPSS 22.0 software (SPSS Inc., Chicago, United States). The difference was considered statistically significant at the level of *P* < 0.05. General linear model analysis was carried out to explore the main influences of eO_3_ and rice cultivars on the soil nematode community. Pearson correlation analysis was used to investigate the relationship between environmental factors and soil nematodes.

To determine the relationship between soil microbial community and soil environmental parameters, redundancy analysis (RDA) was performed by the ‘vegan' package in R. The non-metric multidimensional scaling analysis (NMDS) was carried out to reveal the distribution pattern and factors influencing soil nematode community under different treatments.

Structural equation modeling analysis (SEM) was carried out to explore the effects of eO_3_ and rice cultivars on soil nematodes using AMOS (Amos Development Corporation, Chicago, IL, USA, Version 24.0). Before the SEM, we reduced the dimensionality of variables for soil abiotic factors (pH, NO${}_{3}^{-}$-N, NH${}_{4}^{+}$-N, DOC, and DON), biotic factors (plant, MBC, and MBN), and the dynamics of soil nematodes at different trophic groups through principal component analysis (PCA) (Chen et al., 2013).

The possible pairwise Spearman's rank correlations of nematode community were calculated by the ‘corr.test' function in the ‘psych' package in R. The connections represented significant (*P* ≤ 0.05) and strong (*R*^2^ ≥ 0.6) correlations. A Gephi v.0.9.2 was carried out to visualize the correlation network of soil nematode community (Bastian et al., 2009).

## Results

### Variations of Edaphic Properties

Elevated ozone reduced soil DOC and DON by 17.5 and 40.7%, respectively, since there was no significant interaction between ozone and cultivar. However, eO_3_ and rice cultivars did not significantly affect soil pH, NH${}_{4}^{+}$-N, or NO${}_{3}^{-}$-N content (Table 1). The significant interactive effect of eO_3_ and rice cultivars was observed for MBN (*P* = 0.027): eO_3_ only decreased MBN for Shanyou 63.

**Table 1:** Changes in soil variables under elevated ozone conditions.

**Cultivar**	**Treatment**	**pH**	**DOC**	**DON**	**MBC**	**MBN**	**NO${}_{3}^{-}$-N**	**NH${}_{4}^{+}$-N**
			**mg kg^**−1**^**	**mg kg^**−1**^**	**mg kg^**−1**^**	**mg kg^**−1**^**	**mg kg^**−1**^**	**mg kg^**1**^**
Shanyou 63	CK	7.39 ± 0.06	66.98 ± 13.89a	18.87 ± 5.90a	515.06 ± 121.92	89.52 ± 24.65a	41.64 ± 24.57	20.35 ± 5.02
	eO_3_	7.40 ± 0.07	53.64 ± 6.41ab	12.17 ± 0.45ab	619.69 ± 49.16	53.58 ± 12.14b	53.79 ± 26.83	18.28 ± 3.64
Wuyujing 3	CK	7.35 ± 0.05	59.15 ± 6.09ab	14.18 ± 4.07ab	657.05 ± 93.39	49.32 ± 8.52b	39.68 ± 11.00	16.93 ± 2.34
	eO_3_	7.37 ± 0.06	50.36 ± 1.69b	7.44 ± 5.40b	561.22 ± 48.65	59.99 ± 8.27b	58.80 ± 10.61	21.93 ± 1.49
O_3_ effect^#^		0.20	-17.54	-40.66	0.75	-18.20	38.46	7.83
Cultivar effect*		-0.47	-9.21	-30.37	7.36	-23.60	3.19	0.59
O_3_		0.891	0.049	0.032	0.930	0.182	0.207	0.479
Cultivar		0.446	0.279	0.107	0.415	0.086	0.897	0.954
O_3_×Cultivar		0.420	0.648	0.993	0.073	0.027	0.767	0.109

Notes: #, main effects of elevated O_3_ calculated as ((eO_3_/CK) – 1) × 100; ^*^, main effects of cultivars calculated as ((Wuyujing 3/Shanyou 63) – 1) ×100.

### The Abundance and Diversity of Soil Nematodes

There were 38 genera of soil nematodes across all of the treatments. The herbivore nematodes (*Helicotylenchus* and *Meloidogyne*) were the dominant genera among all treatments (Table S1). Elevated ozone reduced the total nematode abundance by 28.4% (*P* = 0.052) (Table 2, Table S2), but had no effects on that of different trophic groups or the diversity of soil nematode community (Table S2, Figure S1). By contrast, eO_3_ significantly decreased the abundances of c-p 1+2 groups (*P* = 0.067), rather than that of c-p 3 and c-p 5 in both cultivars (Table S2). Shanyou 63 reversed the negative impacts of eO_3_ on the abundance of c-p 4, as indicated by the significant interaction between eO_3_ and cultivars (*P* = 0.034). Similarly, eO_3_ increased the abundance of bacterivores belonging to c-p 4 (i.e., BF4) for Shanyou 63, rather than Wuyujing 3 (Table 2). Elevated ozone had no significant impact on the relative abundance of nematode functional groups for both cultivars, while slightly increasing that of c-p 4 for Shanyou 63 (Figure S2).

**Table 2:** Changes in the abundance of soil nematodes (individuals per 100 g dry soil) under elevated ozone conditions.

**Nematode groups**	**Shanyou 63**		**Wuyujing 3**		**ANOVA Result**	
	**CK**	**eO3**	**CK**	**eO3**	***P* value**	***F* value**
Total	123.22 ± 19.49	93.04 ± 26.74	122.24 ± 36.98	82.83 ± 18.24	0.224	1.81
Trophic groups						
BF	26.28 ± 4.52	26.74 ± 16.17	17.5 ± 8.26	14.07 ± 11.68	0.443	0.99
FF	13.73 ± 6.10	7.45 ± 8.09	7.16 ± 1.99	4.71 ± 5.01	0.274	1.55
PF	72.13 ± 17.59	50.19 ± 17.69	89.06 ± 29.75	53.90 ± 37.76	0.320	1.37
OP	11.09 ± 5.63	8.66 ± 4.55	8.52 ± 5.63	10.15 ± 6.67	0.932	0.14
Functional groups						
BF1	10.96 ± 6.92	3.51 ± 2.70	4.94 ± 5.12	5.74 ± 5.49	0.392	1.13
BF2	10.95 ± 5.79	7.30 ± 6.70	5.84 ± 2.83	6.25 ± 6.58	0.692	0.50
BF3	3.48 ± 1.90	3.36 ± 4.08	4.48 ± 0.79	1.85 ± 1.23	0.613	0.63
BF4	0.89 ± 1.55*b*	12.56 ± 8.37*a*	2.24 ± 0.77*b*	0.23 ± 0.39*b*	0.024	5.49
FF2	13.73 ± 6.84	7.45 ± 5.87	6.27 ± 0.76	3.91 ± 4.36	0.180	2.09
FF3	0 ± 0	0 ± 0	0.89 ± 1.54	0.80 ± 0.87	0.474	0.92
PF2	25.79 ± 18.57	12.68 ± 2.39	10.77 ± 2.40	7.39 ± 7.33	0.208	1.90
PF3	45.63 ± 7.08	37.51 ± 17.38	77.85 ± 30.87	45.94 ± 36.09	0.295	1.47
PF4	0 ± 0	0 ± 0	0.44 ± 0.77	0 ± 0	0.441	1.00
PF5	0.70 ± 1.22	0 ± 0	0 ± 0	0.57 ± 0.99	0.592	0.67
OP3	0 ± 0	0 ± 0	0.44 ± 0.77	0 ± 0	0.441	1.00
OP4	2.30 ± 2.14	1.61 ± 1.41	0.45 ± 0.78	0 ± 0	0.215	1.86
OP5	8.79 ± 3.57	7.06 ± 3.26	7.63 ± 5.12	10.15 ± 6.67	0.867	0.24

*Note: TN, total nematodes; BF, bacterivores; FF, fungivores; PF, herbivores; OP, omnivores-predators*.

### Soil Nematode Community

**Figure 1:**
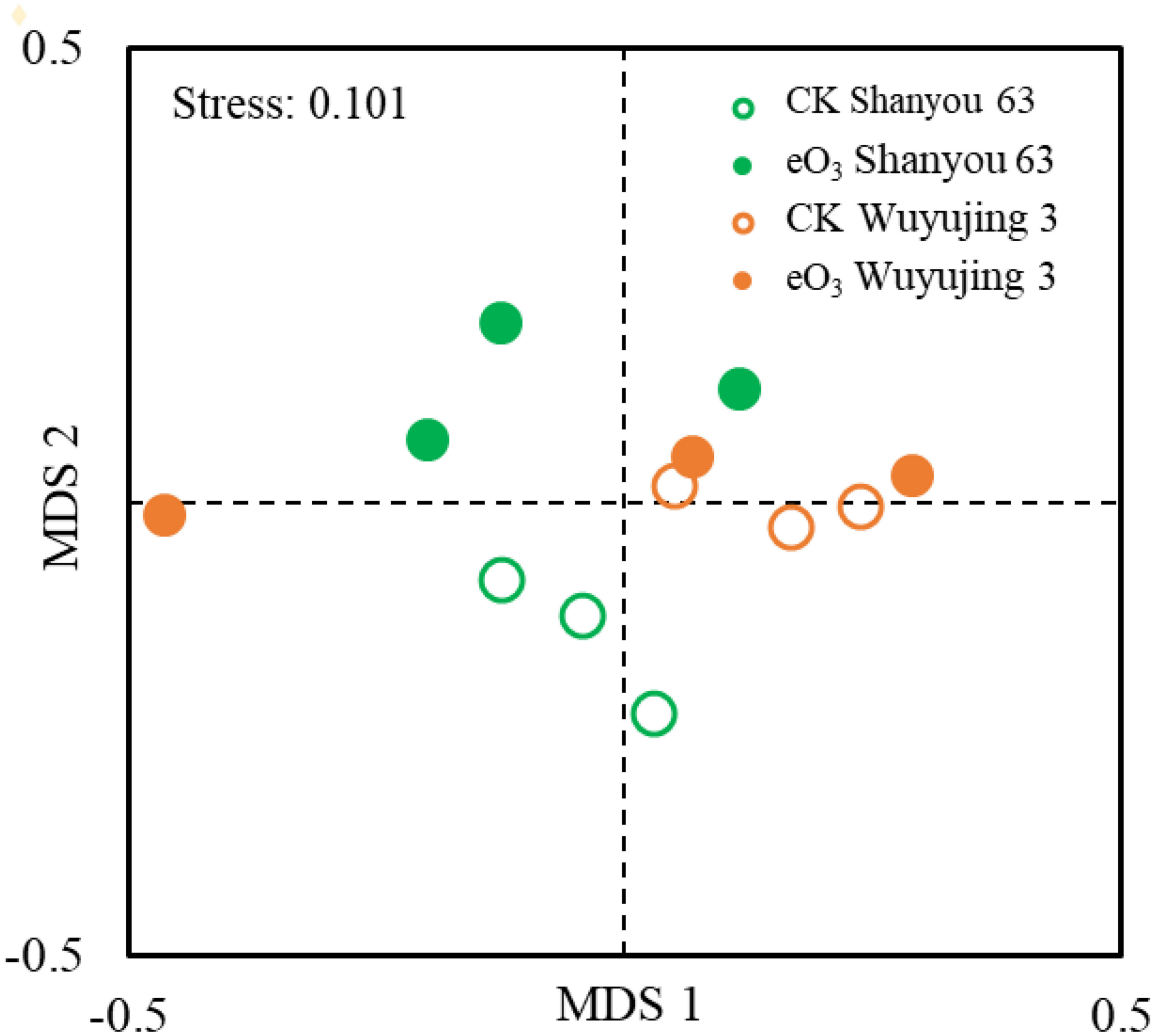
Non-multidimensional scaling (NMDS) analysis of the nematode community under elevated ozone.

**Figure 2:**
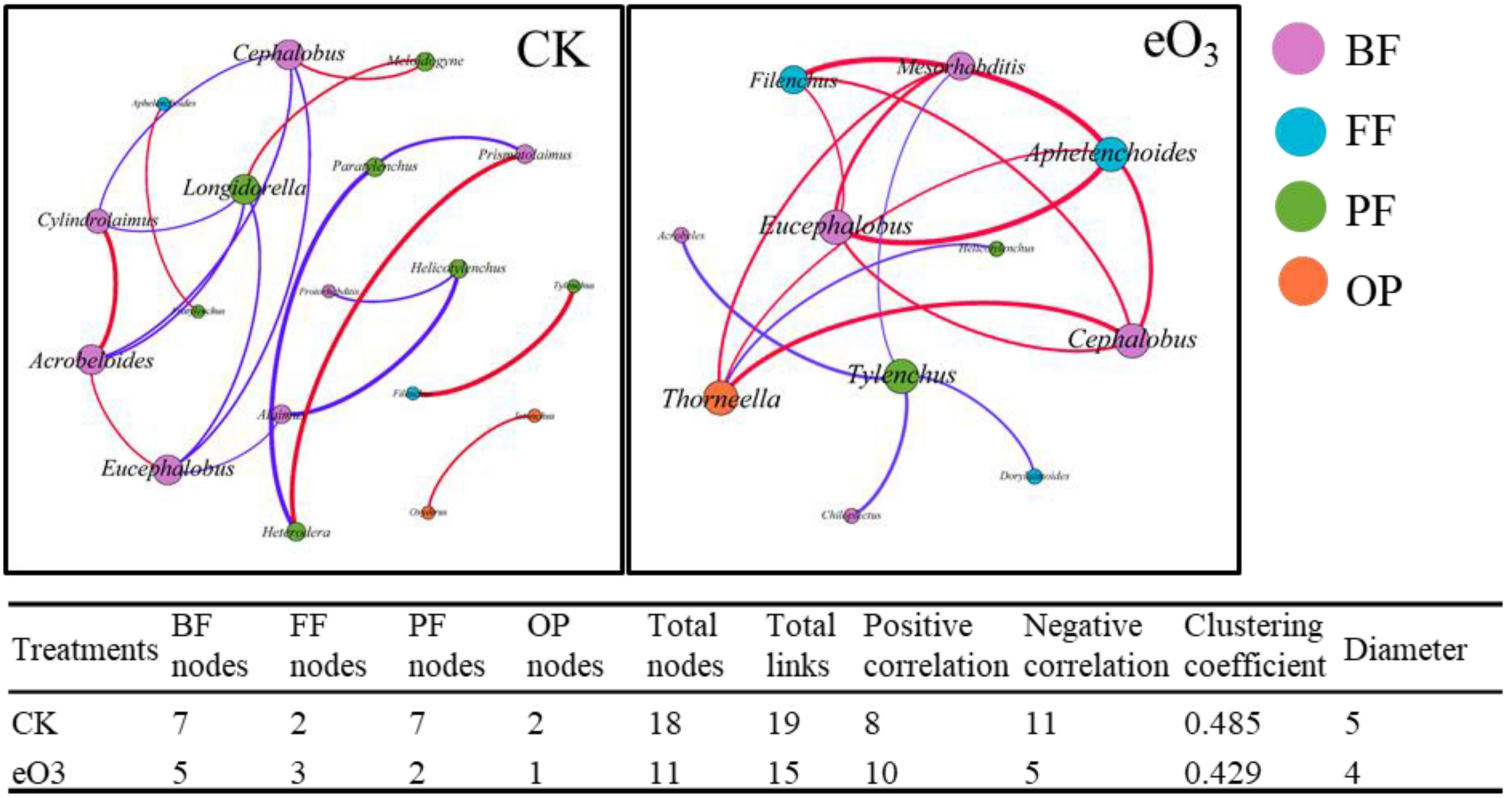
The complexity and interactions of soil nematode community under elevated ozone. The connections represented strong (*R*^2^ > 0.6) and significant (*P* < 0.05) correlations. The size of each node was proportional to the number of connections (degrees). Red lines represented significantly positive and purple lines represent significantly negative correlations. Different colors of nodes represented nematode trophic groups.

The effects of eO_3_ and rice cultivars were evaluated by NMDS (Figure 1). Soil nematode community in CK was clearly separated from those under eO_3_ in Shanyou 63, but not for Wuyujing 3, indicating that the ozone effect was largely dependent on rice cultivars (Figure 1). Network analysis was used to visualize the structural complexity of the soil nematode community, which showed that the networks were simplified by eO_3_ (Figure 2). In the network, most nodes were derived from BF and PF, which showed decreasing node numbers under eO_3_. Total nodes in the network were minimized by eO_3_, and the connectivity in the eO_3_ treatment was 21.1% lower than CK.

### Relationships Between Soil Nematodes and Soil Properties

**Figure 3:**
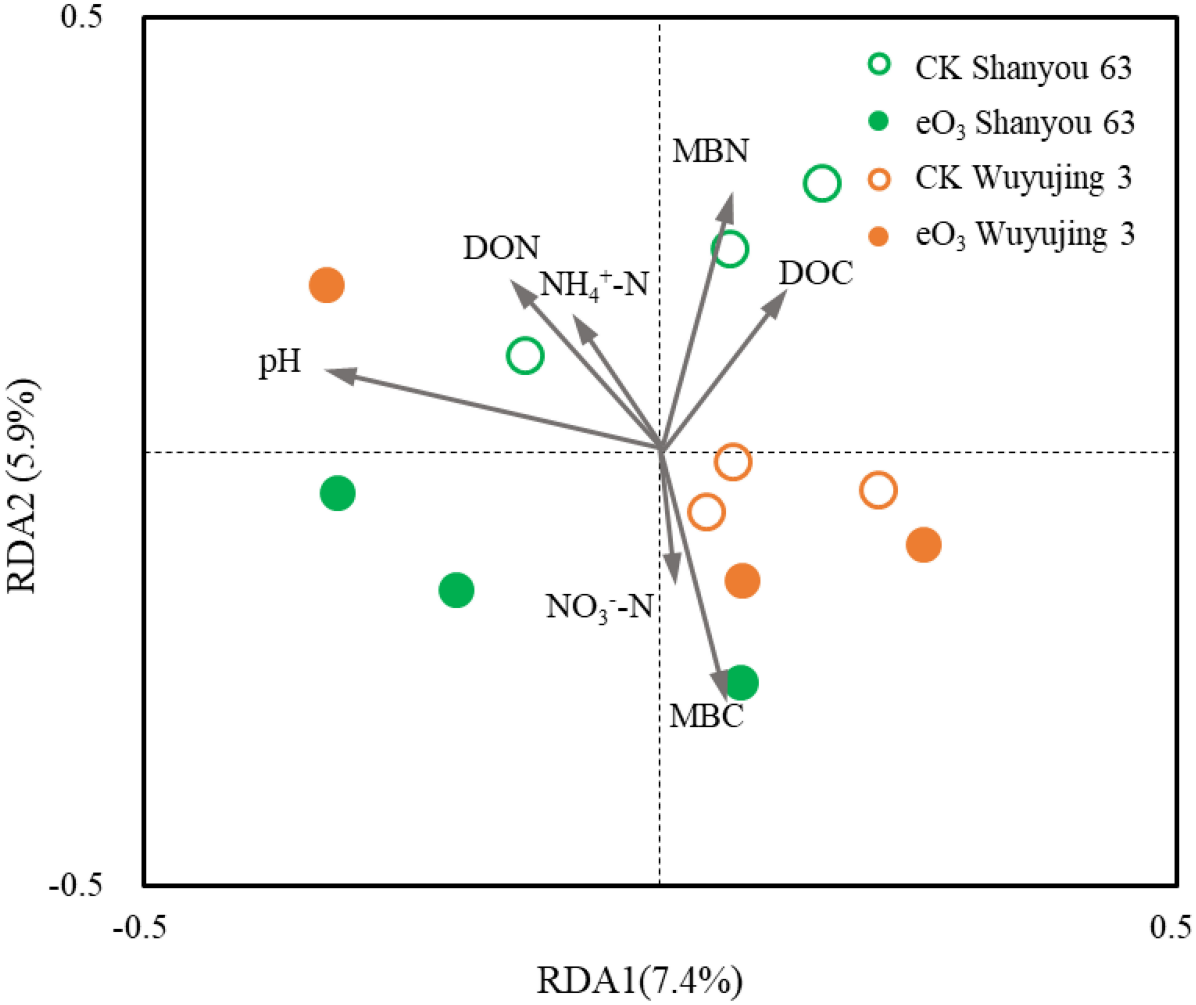
Redundancy analysis (RDA) of the relationships between soil properties and soil nematode community under elevated ozone.

**Figure 4:**
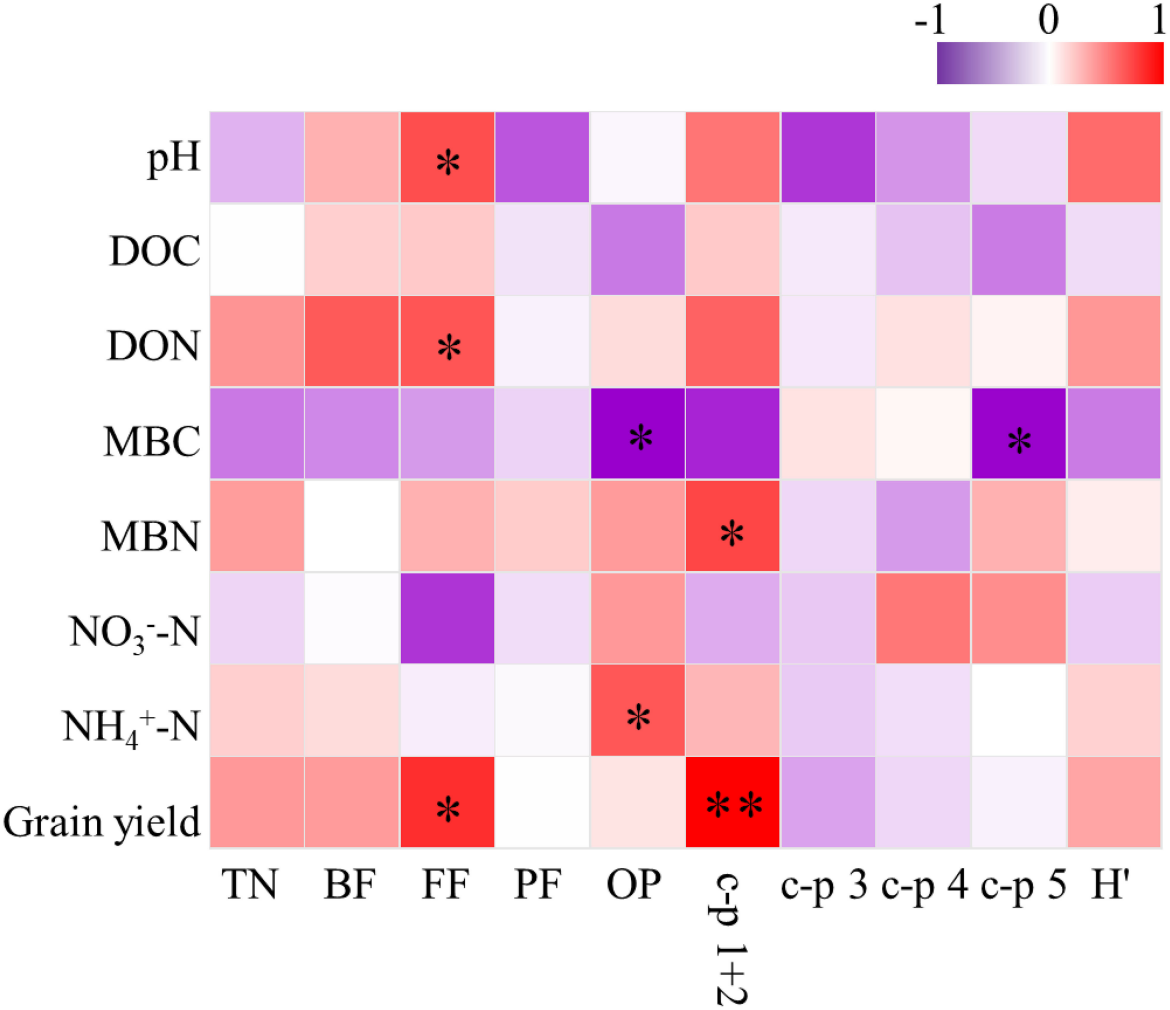
Correlations between soil properties, grain yield and soil nematode community under elevated ozone. DOC, dissolved organic carbon; DON, dissolved organic nitrogen; MBC, microbial biomass carbon; MBN, microbial biomass nitrogen; TN, total nematodes; BF, bacterivores; FF, fungivores; PF, plant parasites; OP, omnivorous-predatory; H′, Shannon-Wiener diversity index. * The asterisk represents a significant correlation at the 0.05 probability level.

Soil pH was the most important parameter contributing to the changes in soil nematode community, followed by soil MBC and MBN (Figure 3). Correlation analysis revealed that the abundance of FF was positively correlated with soil pH, DON, and grain yield (Figure 4). The abundance of OP was positively correlated with soil NH${}_{4}^{+}$-N but negatively related to soil MBC. There was a significant positive correlation between the c-p 1+ 2 groups and MBN as well as grain yield. The abundance of the c-p 5 groups was significantly and negatively correlated with MBN.

**Figure 5:**
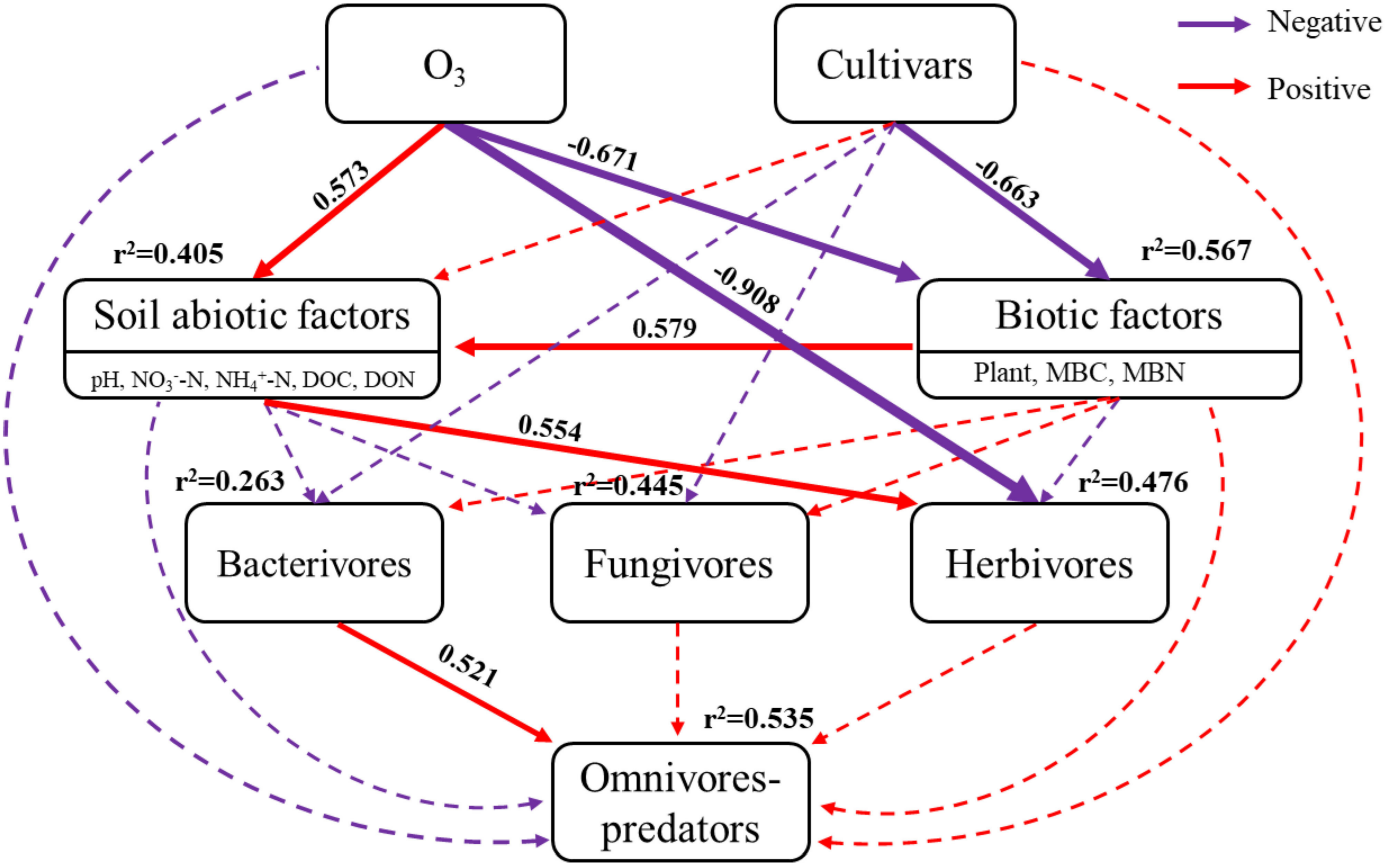
Structural equation models (SEM) analysis of elevated ozone and rice cultivars on soil nematodes. The results of the model fitting: Chi-square (χ^2^) = 4.635, *P* = 0.704, df = 7, CFI (comparative fit index) = 1, RMSEA (root square mean error of approximation) < 0.001. Red and purple arrows showed positive and negative effects, while solid arrows and dashed arrows showed the directions and effects that were significant (*P* < 0.05) and non-significant (*P* > 0.05), respectively.

The Chi-square (χ^2^) test of SEM fit (χ^2^ = 4.635, *P* = 0.704, df = 7), CFI (comparative fit index) = 1.000, and RMSEA (root square mean error of approximation) < 0.001, indicated that the SEM was of excellent fit (Figure 5). Elevated ozone positively correlated with soil abiotic factors (covariance coefficient = 0.573), but negatively correlated with biotic factors (covariance coefficient = −0.671). Rice cultivars only negatively correlated with biotic factors (covariance coefficient = −0.663). Therefore, eO_3_ and cultivars explained 40.5 and 56.7% of the total variances in abiotic factors, and biotic factors, respectively. Elevated ozone, cultivars, soil abiotic factors, and biotic factors pathways explained 26.3, 44.5, and 47.6% of the total variances in bacterivores, fungivores, and herbivores, respectively. Interestingly, herbivores were directly related to eO_3_ (covariance coefficient = −0.908) and statistically associated with soil abiotic factors (covariance coefficient = 0.554). The model explained 53.5% of the variance in omnivores-predators, which positively correlated with bacterivores (covariance coefficient = 0.521).

## Discussion

### Elevated Ozone Reduced the Abundance and Community Complexity of Soil Nematodes

We found that eO_3_ reduced the total abundance of soil nematodes (Table 2). This result was consistent with several previous studies, which found that the abundance of soil nematodes was significantly decreased by eO_3_ (Bao et al., 2014; Li et al., 2015). This could be explained by the fact that ozone is an effective biocide, and eO_3_ can inhibit the biomass and reproduction of soil bacteria and fungi (Kim et al., 1999; Kanerva et al., 2008). As a consequence, the reduction in bacterial and fungal biomass would affect the soil food web and the abundance of soil nematodes (Zhang et al., 2021).

The SEMs results showed that eO_3_ directly and negatively correlated with herbivores (Figure 5), indicating that eO_3_ directly inhibited the abundance of soil nematodes. Consistent with Qiu et al. (2009), it was found that the root-knot nematodes were reduced by 24 and 68%, and the free-living nematodes were decreased by 19 and 52% at the ozone concentration of 50 and 250 kg hm^−2^, respectively. Previous studies have confirmed that ozone is an active oxidant with biocidal characteristics, which could directly kill soil nematodes (Mitsugi et al., 2017; Veronico et al., 2017). Therefore, the survival rate of natural nematodes can be directly minimized as the application rate of ozone increases (Mitsugi et al., 2017).

In addition, the impact of eO_3_ on plant growth could indirectly inhibit the activity and reproduction of the soil nematode community (Wang et al., 2021; Zhang et al., 2021). Ozone as a phytotoxic compound, can inhibit plant growth and decrease root exudates and carbon allocation (Hu et al., 2018), resulting in reduced resource availability for the soil microbial community. It has been acknowledged that ozone can decrease the transportation of carbohydrates to rhizodeposition, which inhibited the reproduction of arbuscular mycorrhizal fungi, and thus provided fewer nutrients for soil nematodes (Wang et al., 2011; Zhang et al., 2021). Li et al. (2012) found that DOC content was decreased by eO_3_, and the effect of eO_3_ on the soil food web was associated with soil carbon and nitrogen contents. In the present study, we found that eO_3_ decreased the contents of DOC and DON (Table 1), which correlated to the abundances of FF and PF (Figure 3). Furthermore, the crop yields under eO_3_ were observed to be reduced by 37.8% in the present study (Figure S3). The detrimental effect of eO_3_ on resource partitioning between above- and below-ground may lead to an indirect influence on the stability of the soil nematode community.

Consistent with our first hypothesis, the structural complexity of the soil nematode community was simplified by eO_3_ (Figure 2), indicating that eO_3_ reduced the complexity and interactions of the soil food web. As mentioned above, the abundance of soil nematode was decreased under eO_3_, which was partially ascribed to the reduction in crop production (Figure S3). The variation in plant inputs to the soil under eO_3_ can propagate through carbon resources to alter the complexity and interactions of the soil food web (Chung et al., 2006). Moreover, the declines in root biomass induced by eO_3_ meant fewer resources available for the soil nematode communities (Bao et al., 2014). Accordingly, eO_3_ had a direct and negative impact on soil nutrients such as DOC and DON (Table 1) and subsequently propagated into soil nematode communities, especially herbivores (Figure 5). Given that the network complexity of the feeding links was significantly correlated with the stability and stress tolerance of the soil food web (Beckerman et al., 2006), our findings thus highlighted that eO_3_ went against the stability and interactions of the soil micro-food web.

### Rice Cultivars Altered the Effects of eO_3_ on Functional Groups of Soil Nematodes

Soil nematode functional groups were sensitive to eO_3_ and cultivar effects (Li et al., 2012). Interestingly, the abundance of bacterivores belonging to *K*-strategies (BF4) was increased under eO_3_ (Table 2). The increase in the abundance of bacterivores within *K*-strategies (BF4) groups may be caused by the stability of the bacterial feeding channel under the stress conditions (De Vries et al., 2013). It has been recognized that the bacterial feeding channel (bacteria and bacterivores) is more resilient than other trophic groups of the slowly growing biotas under climate change (De Vries et al., 2013). Our result showed that bacterivores were directly and statistically associated with omnivores-predators (Figure 5), indicating that the soil micro-food web formed a bacterial-dominated channel under eO_3_ in paddy soil.

Our results suggested that rice cultivars altered the adverse effects of eO_3_ on soil nematodes. In favor of our hypothesis, the cultivar of Shanyou 63 reversed the negative impact of eO_3_ on the abundance of soil nematodes belonging to c-p 4 (Table 2). This is consistent with a previous study by Li et al. (2012), who observed that eO_3_ reduced the abundance of bacterivores in the ozone-sensitive wheat but increased it in the ozone-tolerant wheat. Rice has been considered vulnerable to eO_3_ (Feng and Kobayashi, 2009). The response of rice yield to eO_3_ showed varietal difference (Pang et al., 2009), which indirectly altered the effect of eO_3_ on soil nematodes through changes in the input of nutrient resources. Therefore, the negative effect of eO_3_ on the soil nematode community could be weakened under the hybrid Indica cultivar in paddy soil.

Crop cultivars played an essential role in determining the response of soil nematode community to eO_3_ (Li et al., 2012; Feng et al., 2017). The community composition of soil nematodes responded differently to specific plants under eO_3_ (De Deyn et al., 2004). In this study, rice cultivars significantly influenced the nematode abundance of c-p 1+2. In particular, the nematode abundance of c-p 1+2 groups in Wuyujing 3 was decreased by 44.7% compared with Shanyou 63 (Table 2). Plants can influence soil biota through changes in the inputs of root exudates and plant litter (Zhang et al., 2019). Previous studies have revealed that the aboveground biomass, yield, and harvest index of Shanyou 63 were higher than that of Wuyujin 3 (Xu et al., 2007; Pang et al., 2009). Data from the same experiment showed that the grain yield of Shanyou 63 was 31.9% higher than that of Wuyujing 3 (Figure S3). The changes in specific soil nematode groups could therefore depend on plant inputs of soil food resources and energy, and the drop in crop biomass could directly result in the decreased abundance of soil nematodes under climate change.

In this study, eO_3_ increased the abundances of c-p 4 groups and bacterivores belonging to *K*-strategies in Shanyou 63, rather than Wuyujing 3, indicating that the hybrid Indica cultivar mitigated the negative impact of eO_3_ on the functional groups of soil nematode community. This finding provided important insights for the adaptation of soil food web and ecosystem functions to climate change in the agricultural ecosystem. Considering that the stress derived from eO_3_ on soil nematodes and food production continues to increase in near future (Ainsworth, 2017). It is an essential task to develop new rice cultivars and benefit from the positive aspects of cultivars with the capacity for restraining the adverse effects of climate change (i.e., eO_3_) on soil health and ecosystem functions (Ortiz et al., 2008; Wang et al., 2019).

## Conclusion

This study demonstrated that eO_3_ significantly reduced the total abundance and simplified the structural complexity of the soil nematode community. However, the hybrid Indica cultivar could tradeoff the negative impact of eO_3_ on the abundance of bacterivores belonging to *K*-strategies, suggesting that the hybrid Indica cultivar may alleviate the threat of eO_3_ to the soil micro–food web. This study revealed that the soil nematode community was altered by eO_3_, but the variations of nematode functional groups were dependent on rice cultivars. Our study highlighted that the breeding and biotechnological approaches could be valuable approaches to tradeoff the adverse impacts of eO_3_ on the soil nematode community, and improve the potential feedback of soil micro-food web in agricultural ecosystems.

## Data Availability Statement

The original contributions presented in the study are included in the article/Supplementary Material, further inquiries can be directed to the corresponding author/s.

## Author Contributions

JW and YT analyzed the data. YT and YS performed the laboratory work. JW and XS drafted the manuscript. XS and GZ contributed ideas to the study and revised the first draft. All authors contributed to the article and approved the submitted version.

## Funding

The research was supported by the National Natural Science Foundation of China (Grant Nos. 42077209, 31901165, 32071631, and 41907022), the Sino-German Mobility Programme (M-0105), the Open Project of Jiangsu Key Laboratory of Crop Genetics and Physiology (YCSL202004), and Natural Science Foundation of Fujian Province, China (Grant No. 2020J01186), and the Open Fund of Key Laboratory of Agrometeorology of Jiangsu Province (JKLAM2001). The design of the study and collection of data was supported by the Startup Foundation for Introducing Talent of Nanjing University of Information Science and Technology (003035), Nanjing, China.

## Conflict of Interest

The authors declare that the research was conducted in the absence of any commercial or financial relationships that could be construed as a potential conflict of interest.

## Publisher's Note

All claims expressed in this article are solely those of the authors and do not necessarily represent those of their affiliated organizations, or those of the publisher, the editors and the reviewers. Any product that may be evaluated in this article, or claim that may be made by its manufacturer, is not guaranteed or endorsed by the publisher.
