# Corrigendum: Changes in the abundance and community complexity of soil nematodes in two rice cultivars under elevated ozone

**DOI:** 10.3389/fmicb.2022.966819

**Published:** 2022-08-11

**Authors:** Jianqing Wang, Yunyan Tan, Yajun Shao, Xiuzhen Shi, Guoyou Zhang

**Affiliations:** ^1^Key Laboratory for Humid Subtropical Eco-geographical Processes of the Ministry of Education, Fujian Normal University, Fuzhou, China; ^2^School of Geographical Sciences, Fujian Normal University, Fuzhou, China; ^3^Key Laboratory of Agrometeorology of Jiangsu Province, School of Applied Meteorology, Nanjing University of Information Science & Technology, Nanjing, China; ^4^Jiangsu Key Laboratory of Crop Genetics and Physiology, Agricultural College of Yangzhou University, Yangzhou, China

**Keywords:** climate change, elevated ozone, soil health, soil food web, paddy soil, soil fauna, rice varieties

In the original article, there was an error in the order of the affiliations. The correct order appears above.

The authors apologize for this error and state that this does not change the scientific conclusions of the article in any way. The original article has been updated.

## Publisher's note

All claims expressed in this article are solely those of the authors and do not necessarily represent those of their affiliated organizations, or those of the publisher, the editors and the reviewers. Any product that may be evaluated in this article, or claim that may be made by its manufacturer, is not guaranteed or endorsed by the publisher.

