# Supplementary material for: Changes in the Abundance and Community Complexity of Soil Nematodes in Two Rice Cultivars Under Elevated Ozone
Source: Front Microbiol. 2022 Jun 9;13:916875. doi: 10.3389/fmicb.2022.916875 (PMC9238508; doi:10.3389/fmicb.2022.916875)
Supplement: Supplementary file 1 [file Data_Sheet_1.docx]

**Table S1** The proportional contribution (%) of soil nematodes to the nematode assemblage under elevated ozone.

| Feeding type | Genus | c-p value | Shanyou 63 | |  | Wuyujing 3 | |
| --- | --- | --- | --- | --- | --- | --- | --- |
|  |  |  | CK | eO_3_ |  | CK | eO_3_ |
| Bacterivores | *Protorhabditis* | 1 | 3.70 | 1.90 |  | 0.31 | 0.57 |
|  | *Mesorhabditis* | 1 | 5.92 | 2.24 |  | 3.34 | 6.84 |
|  | *Monhystera* | 1 | 0.00 | 0.00 |  | 0.00 | 0.93 |
|  | *Acrobeles* | 2 | 0.00 | 0.71 |  | 0.31 | 2.33 |
|  | *Anaplectus* | 2 | 0.00 | 0.00 |  | 0.00 | 0.93 |
|  | *Chiloplectus* | 2 | 0.00 | 0.71 |  | 0.31 | 1.16 |
|  | *Acrobeloides* | 2 | 3.56 | 1.66 |  | 1.44 | 0.81 |
|  | *Eucephalobus* | 2 | 6.54 | 3.32 |  | 2.06 | 2.09 |
|  | *Cephalobus* | 2 | 0.00 | 0.95 |  | 0.62 | 0.93 |
|  | *Plectus* | 2 | 0.00 | 0.00 |  | 0.56 | 0.93 |
|  | *Cylindrolaimus* | 3 | 3.56 | 0.00 |  | 0.56 | 0.00 |
|  | *Prismatolaimus* | 3 | 0.00 | 3.08 |  | 3.31 | 2.31 |
|  | *Alaimus* | 4 | 0.93 | 12.75 |  | 1.81 | 0.24 |
| Fungivores | *Aphelenchus* | 2 | 0.00 | 0.00 |  | 0.32 | 0.57 |
|  | *Aphelenchoides* | 2 | 8.81 | 4.28 |  | 1.81 | 1.85 |
|  | *Ditylenchus* | 2 | 0.17 | 1.90 |  | 0.00 | 0.00 |
|  | *Filenchus* | 2 | 4.21 | 4.14 |  | 3.51 | 3.35 |
|  | *Dorylaimoides* | 4 | 0.00 | 0.00 |  | 0.63 | 1.16 |
| Herbivores | *Tylenchus* | 2 | 9.51 | 12.77 |  | 6.37 | 7.86 |
|  | *Paratylenchus* | 2 | 6.34 | 0.00 |  | 3.06 | 0.48 |
|  | *Psilenchus* | 2 | 0.00 | 0.00 |  | 0.32 | 0.00 |
|  | *Helicotylenchus* | 3 | 13.24 | 13.26 |  | 10.79 | 9.22 |
|  | *Juv. Heterodera* | 3 | 0.00 | 3.65 |  | 2.68 | 1.43 |
|  | *Pratylenchus* | 3 | 4.66 | 4.80 |  | 3.01 | 5.58 |
|  | *Juv. Meloidogyne* | 3 | 21.16 | 18.78 |  | 44.05 | 34.31 |
|  | *Longidorella* | 3 | 0.00 | 0.00 |  | 0.32 | 0.00 |
|  | *Hirschmanniella* | 4 | 0.00 | 0.00 |  | 1.87 | 0.00 |
|  | *Xiphinema* | 5 | 0.00 | 0.00 |  | 0.00 | 0.93 |
|  | *Longidorus* | 5 | 0.17 | 0.00 |  | 0.00 | 0.00 |
| Omnivores-predators | *Tripyla* | 3 | 0.00 | 0.00 |  | 0.32 | 0.00 |
|  | *Thorneella* | 4 | 2.22 | 0.00 |  | 0.00 | 0.00 |
|  | *Pungentus* | 4 | 0.93 | 0.95 |  | 0.00 | 0.00 |
|  | *Eudorylaimus* | 4 | 0.17 | 0.00 |  | 0.31 | 0.00 |
|  | *Iotonchus* | 4 | 0.00 | 0.71 |  | 0.00 | 0.00 |
|  | *Prodorylaimus* | 5 | 0.00 | 0.95 |  | 0.00 | 0.93 |
|  | *Nygolaimus* | 5 | 0.93 | 0.00 |  | 0.00 | 0.00 |
|  | *oxydirus* | 5 | 5.33 | 6.47 |  | 5.72 | **11.33** |
|  | *Discolaimus* | 5 | 0.00 | 0.00 |  | 0.31 | 0.00 |
| Number of genus | | | 20 | 21 |  | 28 | 25 |
| Number of dominated genus | | | 2 | 4 |  | 2 | 1 |

Note: Dominant genera accounted for more than 10% of total nematodes.

**Table S2** Summary of the analysis of GLMM of soil nematode densities under elevated ozone conditions.

| Source | TN | BF | FF | PF | OP | c-p 1+2 | c-p 3 | c-p 4 | c-p 5 |
| --- | --- | --- | --- | --- | --- | --- | --- | --- | --- |
| O_3_ effect ^#^ | -28.35 | -6.78 | -36.94 | -36.04 | -4.07 | -39.22 | -32.78 | 110.63 | 3.87 |
| Cultivar effect * | -5.17 | -40.46 | -49.12 | 18.98 | -5.46 | -44.66 | 45.10 | -70.92 | 10.91 |
| O_3_ | 0.052 | 0.821 | 0.263 | 0.101 | 0.906 | 0.067 | 0.202 | 0.326 | 0.959 |
| Cultivar | 0.723 | 0.131 | 0.122 | 0.487 | 0.874 | 0.037 | 0.230 | 0.131 | 0.717 |
| O_3_×Cultivar | 0.77 | 0.767 | 0.654 | 0.7 | 0.553 | 0.154 | 0.433 | 0.034 | 0.507 |

Note: TN, total nematodes; BF, bacterivores; FF, fungivores; PF, herbivores; OP, omnivores-predators. #, main effects of elevated O_3_ calculated as (eO_3_/CK- 1) × 100; *, main effects of cultivars calculated as (Wuyujing 3/Shanyou 63 - 1) × 100.


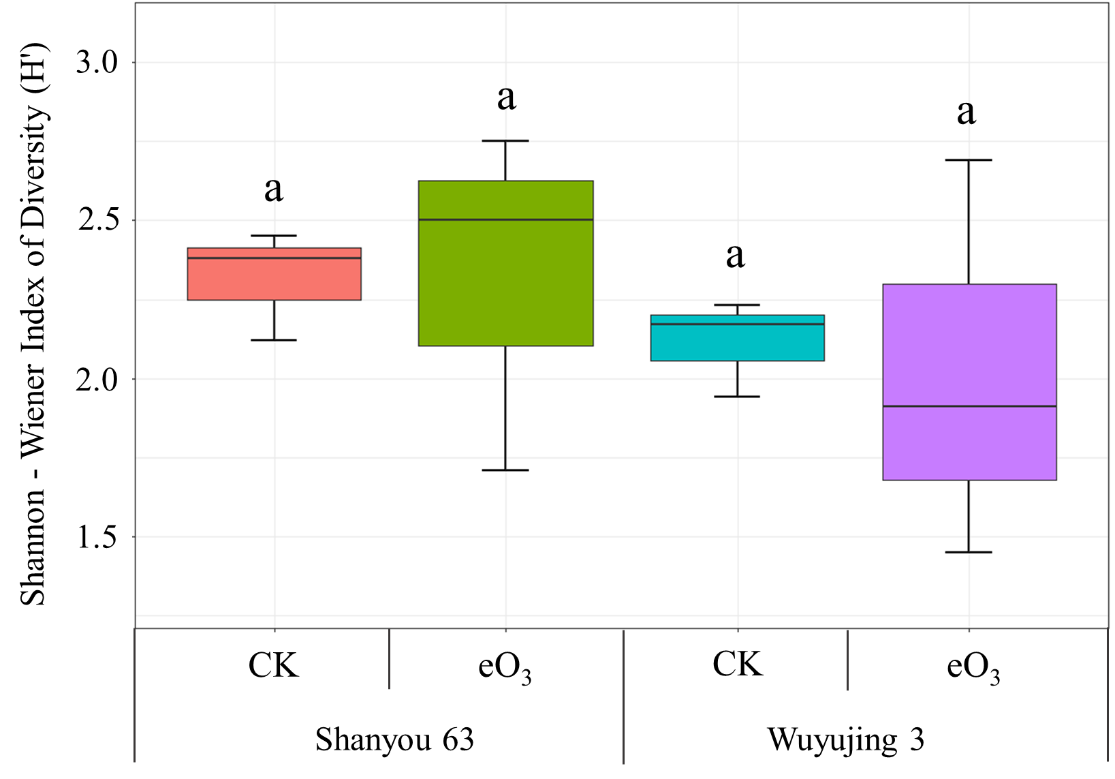
**Fig. S1** Changes in Shannon-Wiener diversity index under elevated ozone.


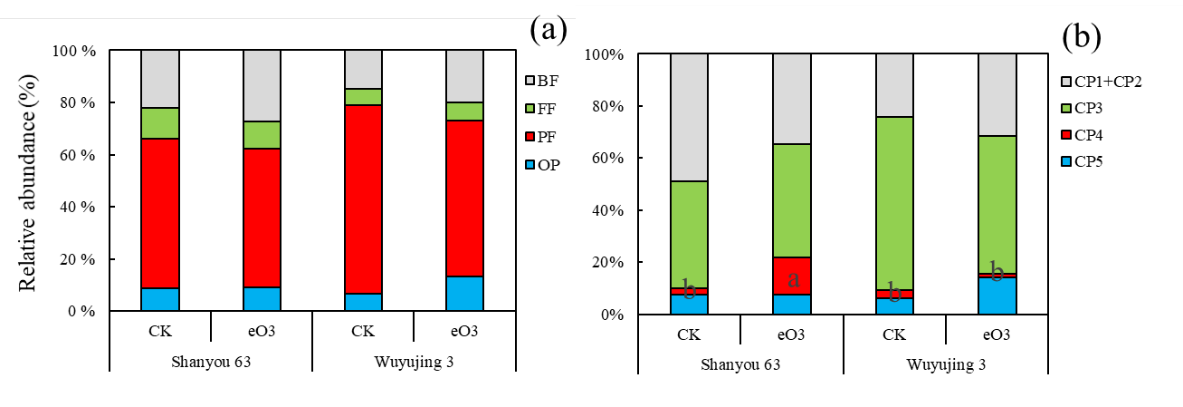
**Fig. S2** Changes in the relative abundance (%) of soil nematode trophic groups and functional groups under elevated ozone.


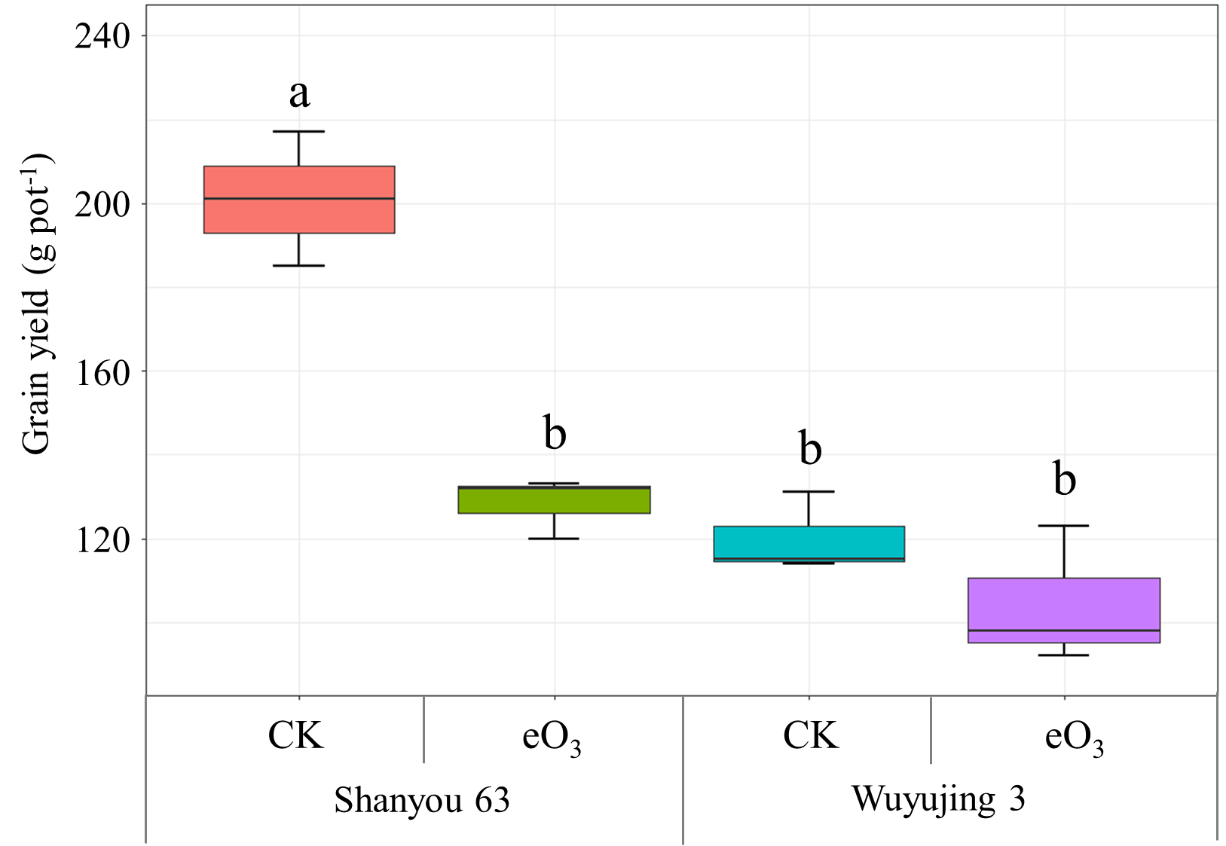
**Fig. S3** Changes in grain yield under elevated ozone.
